# Supplementary material for: Evaluation of the Oral Bacterial Genome and Metabolites in Patients with Wolfram Syndrome
Source: Int J Mol Sci. 2023 Mar 15;24(6):5596. doi: 10.3390/ijms24065596 (PMC10053501; doi:10.3390/ijms24065596)
Supplement: Supplementary file 1 [file ijms-24-05596-s001.zip › Supplementary Table S1.pdf]

Algorithm	p-value	ANOVA (F-value)
Shannon	4.35E-05	12.038
Simpson	5.19E-05	11.789
Fisher	0.00042025	8.9385
Chao1	0.019967	4.1951
ACE	0.042327	3.3444

Supplementary Table S1. Values of all statistical algorithms for assessing alpha diversity analysis between groups.
