# Supplementary material for: Evaluation of the Oral Bacterial Genome and Metabolites in Patients with Wolfram Syndrome
Source: Int J Mol Sci. 2023 Mar 15;24(6):5596. doi: 10.3390/ijms24065596 (PMC10053501; doi:10.3390/ijms24065596)
Supplement: Supplementary file 1 [file ijms-24-05596-s001.zip › Supplementary Table S2.pdf]

Genus	P values	FDR
g__Olsenella	≤ 0.05	≤ 0.05
g__Dialister	≤ 0.05	≤ 0.05
g__Staphylococcus	≤ 0.05	≤ 0.05
g__Campylobacter	≤ 0.05	≤ 0.05
g__Actinomyces	≤ 0.05	≤ 0.05
g__Parvimonas	≤ 0.05	≤ 0.05
g__Bosea	≤ 0.05	≤ 0.05
g__Treponema	≤ 0.05	≤ 0.05
g__Lactobacillus	≤ 0.05	≤ 0.05
g__Selenomonas	≤ 0.05	≤ 0.05
g__Fusobacterium	≤ 0.05	≤ 0.05
g__Leptotrichia	≤ 0.05	≤ 0.05
g__Bifidobacterium	≤ 0.05	≤ 0.05
g__Burkholderia	≤ 0.05	≤ 0.05
g__Ralstonia	≤ 0.05	≤ 0.05
g__Streptococcus	≤ 0.05	≤ 0.05
g__Rothia	≤ 0.05	≤ 0.05
g__Corynebacterium	≤ 0.05	≤ 0.05
g__Cupriavidus	≤ 0.05	≤ 0.05
g__Veillonella	≤ 0.05	≤ 0.05
g__Cellulomonas	≤ 0.05	≤ 0.05
g__Azospira	≤ 0.05	≤ 0.05
g__Tannerella	≤ 0.05	≤ 0.05
g__Neisseria	≤ 0.05	≤ 0.05
g__Xanthomonas	≤ 0.05	≤ 0.05
Not_Assigned	≤ 0.05	≤ 0.05
g__Haemophilus	≤ 0.05	≤ 0.05
g__Mycoplasma	≤ 0.05	0.058133
g__Lachnoanaerobaculum	≤ 0.05	0.075371
g__Prevotella	0.091231	0.1338
g__Atopobium	0.10697	0.15134
g__Enterococcus	0.11007	0.15134
g__Cardiobacterium	0.17429	0.23239
g__Capnocytophaga	0.2557	0.3309
g__Bacteroides	0.35281	0.43266
g__Eikenella	0.354	0.43266
g__Bacillus	0.37202	0.4424
g__Megasphaera	0.41047	0.47528
g__Pseudopropionibacterium	0.49899	0.56296
g__Aggregatibacter	0.5587	0.61457
g__Mogibacterium	0.62307	0.66866
g__Schaalia	0.77898	0.81607
g__Lachnoclostridium	0.8848	0.90537
g__Lautropia	0.99672	0.99672

Supplementary Table S2. Important bacteria identified by univariate analysis.
