# Supplementary figures and images for: Evaluation of the Oral Bacterial Genome and Metabolites in Patients with Wolfram Syndrome
Source: Int J Mol Sci. 2023 Mar 15;24(6):5596. doi: 10.3390/ijms24065596 (PMC10053501; doi:10.3390/ijms24065596)

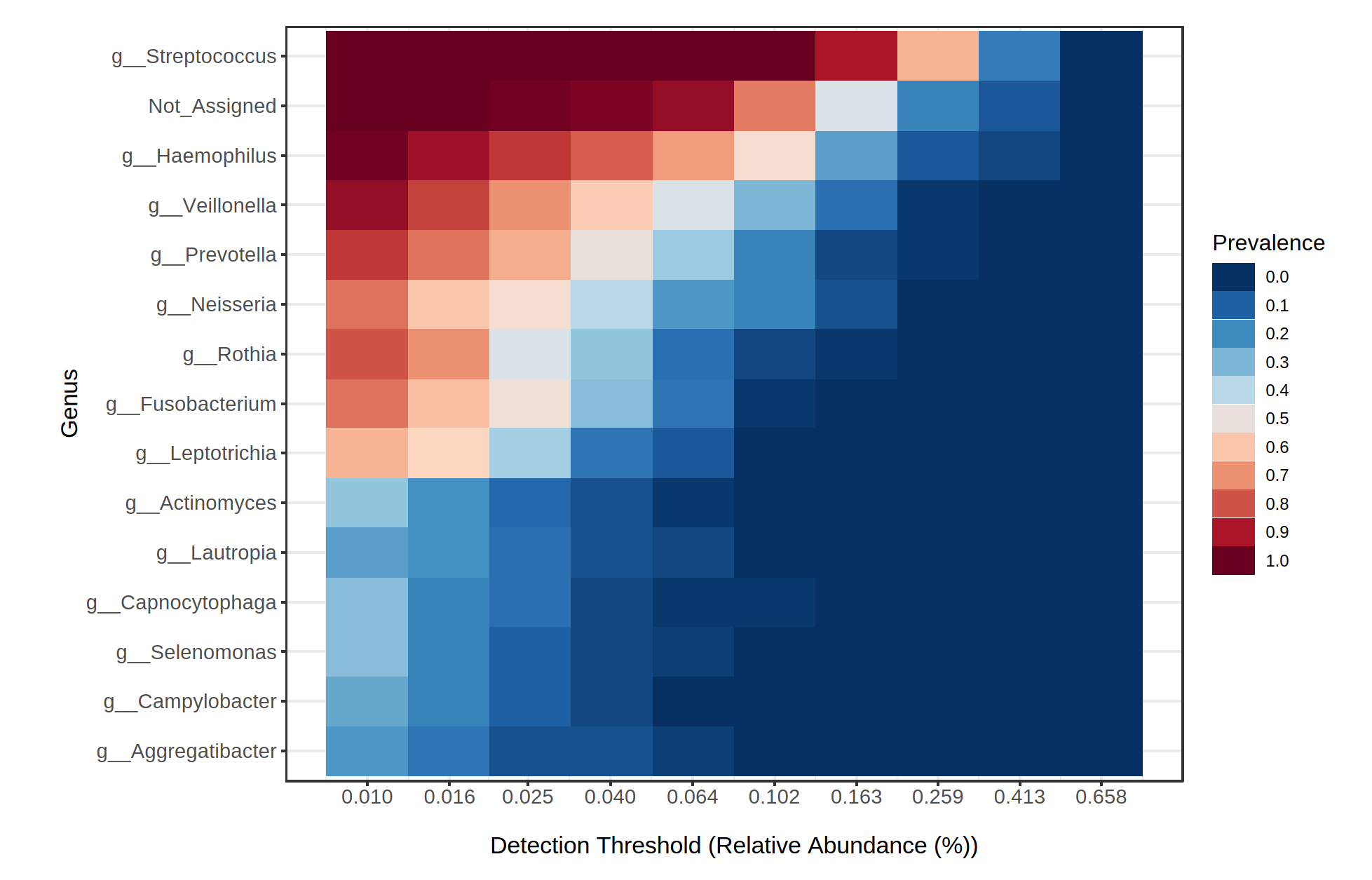

Supplement: Supplementary file 1 [file ijms-24-05596-s001.zip › Supplementary Figure S1.tif]
